# Older Age and Other Protective Factors of Mental Health During the COVID-19 Pandemic in the US

**DOI:** 10.1093/geroni/igab046.2683

**Published:** 2021-12-17

**Authors:** Kelly Smith, Amanda Chappell, Rachael Spalding, Jenna Wilson, Ilana Haliwa, JoNell Strough

**Affiliations:** 1 WVU, morgantown, West Virginia, United States; 2 West Virginia University, Morgantown, West Virginia, United States

## Abstract

Research conducted early in the COVID-19 pandemic (i.e., March 2020) suggested that older adults were less likely to experience negative pandemic-related mental health than younger people. We investigated whether this age-related advantage persisted during the July 2020 spike in COVID-19 cases and investigated links between coping strategies and mental health. We used data from the Understanding America Study (UAS) to conduct a secondary data analysis of participants (N = 5,753) aged 18-110 years (M=46.20, SD=12.88) who completed online self-report surveys twice—once immediately prior to the July spike in cases, and again during the spike. Surveys assessed engagement in protective behaviors (e.g., wearing a mask, washing hands), coping strategies (e.g., calling family/friends, getting extra exercise, meditating), and anxiety and depressive symptoms (using the Patient Health Questionnaire PHQ-4). Multiple regression analyses were used to identify predictors of anxiety and depression during the spike, controlling for previous anxiety and depression, race, ethnicity, income, education, and marital status. Older age and engaging in protective behaviors were associated with less anxiety, whereas coping by calling family/friends was associated with more symptoms, R2 = .71, F(16, 5736) = 885.90, p < .001. Coping by calling and getting extra exercise were associated with fewer depressive symptoms, whereas coping by using social media was associated with more symptoms, R2 = .72, F(16, 5736) = 906.65, p < .001. These findings highlight age as a protective factor for anxiety but not for depression and underscore the importance of social support as a protective factor for mental health.

